# Development and validation of prognostic dynamic nomograms for hepatitis B Virus-related hepatocellular carcinoma with microvascular invasion after curative resection

**DOI:** 10.3389/fonc.2023.1166327

**Published:** 2023-04-19

**Authors:** Shilei Bai, Pinghua Yang, Yanping Wei, Jie Wang, Caixia Lu, Yong Xia, Anfeng Si, Baohua Zhang, Feng Shen, Yexiong Tan, Kui Wang

**Affiliations:** ^1^ Department of Hepatic Surgery II, The Eastern Hepatobiliary Surgery Hospital, Naval Medical University, Shanghai, China; ^2^ Department of Biliary Surgery IV, The Eastern Hepatobiliary Surgery Hospital, Naval Medical University, Shanghai, China; ^3^ International Cooperation Laboratory on Signal Transduction, Eastern Hepatobiliary Surgery Hospital, Naval Medical University, Shanghai, China; ^4^ Laboratory of Signal Transduction, National Center for Liver Cancer, Shanghai, China; ^5^ Department of Hepatic Surgery IV, The Eastern Hepatobiliary Surgery Hospital, Naval Medical University, Shanghai, China; ^6^ Department of Surgical Oncology, Qin Huai Medical District of Jinling Hospital, Nanjing Medical University, Nanjing, China

**Keywords:** microvascular invasion, nomogram, hepatocellular carcinoma, prognosis, hepatitis B virus

## Abstract

**Background and Aim:**

The prediction models of postoperative survival for hepatitis B virus-related hepatocellular carcinoma (HBV-HCC) with microvascular invasion (MVI) have not been well established. The study objective was the development of nomograms to predict disease recurrence and overall survival (OS) in these patients.

**Methods:**

Data were obtained from 1046 HBV-related MVI-positive HCC patients who had undergone curative resection from January 2014 to December 2017. The study was approved by the Eastern Hepatobiliary Surgery Hospital and Jinling Hospital ethics committee, and patients provided informed consent for the use of their data. Nomograms for recurrence and OS were created by Cox regression model in the training cohort (n=530). The modes were verified in an internal validation cohort (n= 265) and an external validation cohort (n= 251).

**Results:**

The nomograms of recurrence and OS based on preoperative serological indicators (HBV-DNA, neutrophil-lymphocyte ratio, a-fetoprotein), tumor clinicopathologic features (diameter, number), surgical margin and postoperative adjuvant TACE achieved high C-indexes of 0.722 (95% confidence interval [CI], 0.711-0.732) and 0.759 (95% CI, 0.747-0.771) in the training cohort, respectively, which were significantly higher than conventional HCC staging systems (BCLC, CNLC, HKLC).The nomograms were validated in the internal validation cohort (0.747 for recurrence, 0.758 for OS) and external validation cohort(0.719 for recurrence, 0.714 for OS) had well-fitted calibration curves. Our nomograms accurately stratified patients with HBV-HCC with MVI into low-, intermediate- and high-risk groups of postsurgical recurrence and mortality. Prediction models for recurrence-free survival (https://baishileiehbh.shinyapps.io/HBV-MVI-HCC-RFS/) and OS (https://baishileiehbh.shinyapps.io/HBV-MVI-HCC-OS/) were constructed.

**Conclusions:**

The two nomograms showed good predictive performance and accurately distinguished different recurrence and OS by the nomograms scores for HBV-HCC patients with MVI after resection.

## Introduction

Hepatocellular carcinoma (HCC) is a prevalent and deadly tumor (1). In East Asia, between 70% and 90% of HCC is related to infection with hepatitis B virus (HBV) (2). The first-line treatment is usually liver resection (LR); however, the long-term outcome after LR remains unsatisfactory due to the high rate of early recurrence (2).

The presence of microvascular invasion (MVI) may significantly influence HCC outcomes following either LR or liver transplantation (LT), increasing the risk of early tumor recurrence (3–6). There appears to be an association between microscopic metastases within the liver related to MVI that is not apparent in other forms of HCC (7–9). It has been suggested that HBV infection promotes angiogenesis by increasing levels of metastasis-associated protein 1 (MTA1) or by suppressing the immune response to migrating tumor cells, resulting in invasion of the vasculature (10, 11).

Several factors have been found to influence outcome in HBV-related MVI-positive HCC following LR. Viral status, tumor clinicopathologic features, the patient’s overall condition (including immune and liver function, and systemic inflammation), surgical factors, and the availability of postoperative adjuvant therapy may potentially contribute to progression and recurrence of the cancer. There is no specific prognostic tool for predicting outcome in HBV-related MVI-positive HCC patients following LR and, although there are several traditional staging systems, such as The American Joint Committee on Cancer (AJCC), Barcelona Clinic Liver Cancer (BCLC), Hong-Kong Liver Cancer staging system (HKLC) and the China Liver Cancer (CNLC) systems, that are used for HCC classification and outcome prediction, there is no single uniformly accepted system (12, 13). Moreover, these criteria vary widely and cannot accurately predict individualized outcomes after surgery in this patient population.

The nomogram, which integrates multiple prognostic factors, is considered reliable for risk quantification and has been used in many cancer varieties (14–18). Nomograms have been developed for the prediction of outcomes of HCC after LR and have greater accuracy than the traditional staging systems (19–21). Nevertheless, there is no specific model for prognosis prediction in HBV-related MVI-positive HCC. Therefore, we used a retrospective analysis of 795 HBV-related MVI-positive HCC patients to develop nomograms for assessing the likelihood of postoperative recurrence and overall survival (OS) to guide clinical decision-making in these patients.

## Materials and methods

### Study design

795 consecutive HBV-related HCC patients were included. The patients had undergone radical LR and had been pathologically confirmed as MVI-positive between January 2014 and December 2017 at Shanghai Eastern Hepatobiliary Surgery Hospital (EHBH). Inclusion criteria were: (I). postoperative pathologically confirmed HCC with positive MVI; (II). patients with Child score of grade A or B; (III). no preoperative antitumor therapy; (IV). the surgical approach was radical LR (R0 resection); (V). no large vessel invasion. Exclusion criteria were: (I). incomplete clinical data; (II). tumor recurrence within 1 month or patient death within 3 months after surgery; (III). patient combined with other tumor history; (IV). bile duct tumor thrombosis. The R function “create Data Partition” was used to divide the 795 patients into training(n=530) and internal validation cohorts(n=265) with a ratio of 2:1 to guarantee random distribution of outcomes between the groups. The training cohort was used for filtering variables and model construction, which were then verified in the validation cohort. A total of 251 patients between January 2014 and December 2017 from Jinling Hospital of Nanjing Medical University served as an external validation cohort. The study was approved by the hospital ethics committee, and patients provided informed consent for the use of their data.

### Preoperative evaluation and LR

Routine preoperative tests included measurement of liver function, hepatitis B and C antigens/antibodies, alpha-fetoprotein (AFP), abdominal ultrasound, abdominal contrast-enhanced magnetic resonance imaging (MRI) or computed tomography (CT), and chest X-ray. Serum HBV deoxyribonucleic acid (HBV-DNA) levels were quantified by polymerase chain reaction (ABI7300 Real-Time PCR System). HCC was diagnosed preoperatively using the criteria of the American Association for the Study of Liver Diseases (AASLD) (22).

All procedures were conventional open surgery with routine use of intraoperative ultrasound, and the extent of resection was determined by the tumor size and location, as well as the general condition of the patient. The shortest distance between the tumor margin and the plane of resection ≥1 cm, depending on the postoperative pathology, was used to define the wide margin (23, 24). The presence of microscopic tumor metastases within the portal or hepatic veins in tissue surrounding the tumor was used to define MVI (25). Tumor differentiation was evaluated using the Edmondson-Steiner system (26). Transcatheter arterial chemoembolization (TACE) was performed on the whole residual liver or the corresponding half of the liver approximately 1 month after LR.

### Follow-up

Follow-up assessments were undertaken every two months in the six-month period following LR and thereafter at three-monthly intervals for 18 months and at six-monthly intervals thereafter. Assessments included testing for the tumor marker AFP in peripheral blood and ultrasound, as well as in enhanced abdominal CT or MRI. Patients on preoperative antiviral treatment (AVT) were advised to continue AVT postoperatively. CT, MRI, or bone scan were conducted if there was suspicion of distant metastases or recurrence. Recurrence was defined as a newly present tumor nodule, either within or outside the liver. Decisions for the management of recurrent tumors were based on recurrence pattern, liver functioning, and the overall condition. The options were re-resection, local ablation, TACE, radiotherapy, systemic therapy or best supportive care, either individually or combined.

The primary endpoint was OS, calculated as the time between the dates of diagnosis and all-cause death, respectively, or last follow-up visit at December 2021. Time to recurrence (TTR) was determined as the time between the dates of surgery and tumor recurrence, respectively.

### Statistical analysis

Continuous variables were compared using t-tests or Mann-Whitney U tests, while categorical variables were compared using χ2 or Fisher exact tests. Survival curves were calculated and compared using the Kaplan-Meier method. Cox regression analyses were used for analysis of prognostic factors.

The nomograms were developed using multivariate analysis of the data of patients in the training. Variables for inclusion were selected using stepwise regression based on the minimum of the Akaike information criterion. Recurrence and OS rates at 1/3/5 years were assessed using nomograms. The concordance index (C-index) calculated by bootstrapping and the area under the time-dependent receiver operating characteristic curve (time-dependent AUC) were used to assess discriminatory capability. Calibration capability was assessed using calibration curves. The C-index and AUC values ranged from 0.5 to 1.0, where 0.5 represents perfectly random and 1.0 represents a perfect fit. Values over 0.7 for both these parameters are considered to have superior predictive power. X-tile was used to select cutoff points for risk stratification (27).

P-values were two-tailed with values < 0.05 considered significant. Data were analyzed in R (http://www.r-project.org/)

## Results

### Baseline patient characteristics

The baseline profiles in the training and validation cohorts are shown in **Table 1**. A total of 1046 HBV-related MVI-positive HCC patients were enrolled, including 530 patients in the training cohort,265 patients in the internal validation cohort and 251 patients in the external validation cohort, respectively. The exclusion criteria are provided in **Supplementary Figure 1**. Of all patients, 932 patients (89.1%) were male, 1024 (97.9%) were Child A grade, while 458 (43.8%) had AFP>400 ng/ml, 325 (31.1%) were HBeAg-positive, and 394 (37.7%) had HBV-DNA>2000 IU/ml. The mean tumor diameter of the patients was 6.7 cm, 144 (13.8%) patients had multiple HCC, 577 (55.2%) patients had a postoperative pathological diagnosis of cirrhosis, and 523 (50.0%) patients underwent postoperative TACE therapy. There were no significant differences in baseline characteristics among the 3 cohorts except that patients in the external validation cohort had a lower ALBI grade II (P=0.011), smaller tumor diameter (P=0.026) and more postoperative TACE rates (P <0.001) when compared with the data of the training and internal validation cohorts.

**Table 1:** Baseline characteristics of patients between training and validation cohort.

Variable	Number (%)/Mean (SD)				*P*
	Total (n=1046)	Training cohort (n=530)	Internal validation cohort (n=265)	External validation cohort (n=251)	
**Age**					0.514
≤60	885(84.6)	454(85.7)	224(84.5)	207(82.5)	
>60	161(15.4)	76(14.3)	41(15.5)	44(17.5)	
**Sex**					0.290
Female	114(10.9)	62(11.7)	22(8.3)	30(12.0)	
Male	932(89.1)	468(88.3)	243(91.7)	221(88.0)	
**Child-Pugh grade**					0.922
A	1024(97.9)	519(97.9)	260(98.1)	245(97.6)	
B	2(2.1)	11(2.1)	5(1.89)	6(2.39)	
**ALBI grade**					**0.011**
I	769(73.5)	373(70.4)	194(73.2)	202(80.5)	
II	277(26.5)	157(29.6)	71(26.8)	49(19.5)	
**TBIL**, μmol/L					0.687
≤17	759(72.6)	387(73.0)	187(70.6)	185(73.7)	
>17	287(27.4)	143(27.0)	78(29.4)	66(26.3)	
**ALB**, g/L					0.815
≤35	68(6.5)	37(6.98)	16(6.04)	15(5.98)	
>35	978(93.5)	493(93.0)	249(94.0)	236(94.0)	
**ALT**, U/L					0.077
≤44	693(66.3)	340(64.2)	172(64.9)	181(72.1)	
>44	353(33.7)	190(35.8)	93(35.1)	70(27.9)	
**PT**, S					0.272
≤13	900(86.0)	447(84.3)	233(87.9)	220(87.6)	
>13	146(14.0)	83(15.7)	32(12.1)	31(12.4)	
**PLT**, *10^9^/ml					0.271
≤100	220(21.0)	112(21.1)	48(18.1)	60(23.9)	
>100	826(79.0)	418(78.9)	217(81.9)	191(76.1)	
**NLR**					0.538
≤2.4	622(59.5)	318(60.0)	162(61.1)	142(56.6)	
>2.4	424(40.5)	212(40.0)	103(38.9)	109(43.4)	
**HBeAg**					0.698
Negative	721(68.9)	363(68.5)	188(70.9)	170(67.7)	
Positive	325(31.1)	167(31.5)	77(29.1)	81(32.3)	
**HBV-DNA**, IU/mL					0.112
≤2000	652(62.3)	314(59.2)	173(65.3)	165(65.7)	
>2000	394(37.7)	216(40.8)	92(34.7)	86(34.3)	
**Antiviral treatment**					0.278
No	765(73.1)	396(74.7)	195(73.6)	174(69.3)	
Yes	281(26.9)	134(25.3)	70(26.4)	77(30.7)	
**AFP**, ng/mL					0.211
≤400	588(56.2)	139 (52.5)	298 (56.2)	151(60.2)	
>400	458(43.8)	126 (47.5)	232 (43.8)	100(39.8)	
**CEA**, ng/mL					0.246
≤10	1033(98.8)	525(99.1)	259(97.7)	249(99.2)	
>10	13(1.2)	5(0.94)	6(2.26)	2(0.80)	
**Ca19-9**, U/mL					0.858
≤37	832(79.5)	418(78.9)	213(80.4)	201(80.1)	
>37	214(20.5)	112(21.1)	52(19.6)	50(19.9)	
**Intraoperative blood transfusion**					0.203
No	884(84.5)	441(83.2)	233(87.9)	210(83.7)	
Yes	162(15.5)	89(16.8)	32(12.1)	41(16.3)	
**Surgical margin**					0.773
Narrow	660(63.1)	332(62.6)	172(64.9)	156(62.2)	
Wide	386(36.9)	198(37.4)	93(35.1)	95(37.8)	
**Tumor diameter**, cm	6.7±3.9	6.7±4.1	7.1±4.0	6.1±3.8	**0.026**
**Tumor number**					0.652
Single	902(86.2)	454(85.7)	233(87.9)	215(85.7)	
Multiple	144(13.8)	76(14.3)	32(12.1)	36(14.3)	
**Tumor capsule**					0.906
Incomplete	858(82.0)	432(81.5)	219(82.6)	207(82.5)	
Complete	188(18.0)	98(18.5)	46(17.4)	44(17.5)	
**Cirrhosis**					0.741
No	469(44.8)	238(44.9)	123(46.4)	108(43.0)	
Yes	577(55.2)	292(55.1)	142(53.6)	143(57.0)	
**Edmondson-Steiner grade**					0.252
I-II	43(4.1)	27(5.1)	9(3.4)	7(2.79)	
III-VI	1003(95.9)	503(94.9)	256(96.6)	244(97.2)	
**Postoperative TACE**					**<0.001**
No	523(50.0)	291(54.9)	162(61.1)	70(27.9)	
Yes	523(50.0)	239(45.1)	103(38.9)	181(72.1)	
**BCLC staging**					0.143
0	34(3.3)	16(3.00)	5(1.89)	13(5.18)	
A	893(85.4)	448(84.5)	236(89.1)	209(83.3)	
B	119(11.4)	66(12.5)	24(9.06)	29(11.6)	

Bold values indicate statistical significance (P < 0.05). TBIL, total bilirubin; ALB, albumin; ALBI, albumin-bilirubin; ALT, Alanine aminotransferase; PT, Prothrombin time; PLT, platelet; NLR, neutrophil‐to‐lymphocyte ratio; HBV-DNA, hepatitis B virus-deoxyribonucleic acid; AFP, alpha fetoprotein; CEA, carcinoembryonic antigen; TACE, Transcatheter arterial chemoembolization, BCLC, Barcelona clinic liver cancer.

### Postoperative TTR and OS in the two cohorts

The postoperative survival curves of the training and validation cohorts are shown in the **Supplementary Figure 2**. The recurrence and OS rates at 1-, 3-, and 5- years were 45.5%, 59.1%, 71.0% vs. 45.7%, 66.8%, 77.2%, and 77.4%, 56.9%, 42.7% vs. 78.0%, 54.4%, 39.3% for the training cohort and internal validation cohort, respectively,. The median TTR was 18.1 months and 16.9 months in the two groups, respectively. For the external validation cohort, the 1-, 3- and 5-year recurrence rates were 37.7%, 62.9% and 68.8%, respectively, and the 1-, 3- and 5-year OS rates were 84.3%, 59.1% and 44.4%, respectively. The median TTR was 19.4 months. Moreover, there was no significant difference in the 1-, 3- and 5-year recurrence and OS among these 3 cohorts.

### Development of TTR and OS nomograms in the training cohort

**Figure 1:**
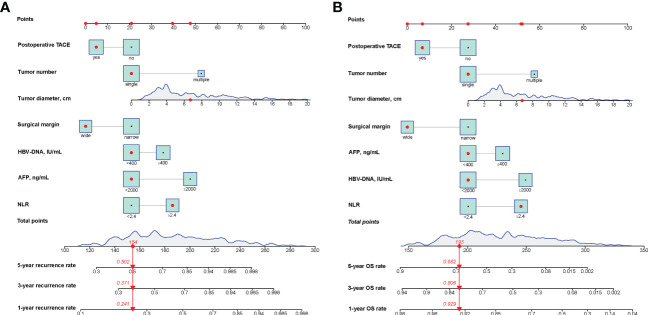
Nomograms for predicting the 1-, 3- and 5-year recurrence **(A)** and OS **(B)** rates in patients with HBV-HCC with MVI. OS, overall survival; HCC, hepatocellular carcinoma; NLR, neutrophilto-lymphocyte ratio; TACE, transcatheter arterial chemoembolisation; AFP, alpha-fetoprotein; HBV-DNA, HBV deoxyribonucleic acid.

Univariate Cox regression showed 15 variables associated with recurrence and 17 associated with survival (**Tables 2**, **3**). After inclusion of these variables in the multivariate analysis, it was found that the neutrophil-lymphocyte ratio (NLR)>2.4, HBV-DNA>2000 IU/ml, AFP>400 ng/ml, narrow surgical margins, tumor diameter, multiple tumors, and the absence of postoperative TACE independently predicted both recurrence and OS (**Tables 2**, **3**). Nomograms were then developed using these results for the prediction of recurrence (**Figure 1A**) and OS rates (**Figure 1B**) at one, three, and five years in HBV-related MVI-positive HCC patients. The nomogram predicted the probabilities by summation of the scores of the individual variables and their location on a scale of the total score.

**Table 2:** Univariate and multivariate cox regression analysis of recurrence in the training cohort.

Variable	Univariate analysis		Multivariate analysis	
	HR (95%CI)	*P*	HR (95%CI)	*P*
**Age**, >60 vs. ≤60 years	0.92(0.69-1.22)	0.558		
**Gender**, male vs. female	1.29(0.93-1.79)	0.120		
**Child-Pugh**, B vs. A grade	1.53(0.81-2.86)	0.189		
**TBIL**, >2000 vs. ≤2000 µmol/L	1.13(0.90-1.41)	0.296		
**ALB**, >35 vs. ≤35 g/L	0.62(0.44-0.89)	**0.009**		
**ALBI**,II vs. I grade	1.52(1.23-1.88)	**<0.001**		
**ALT**, >44 vs. ≤44 U/L	1.20(0.97-1.47)	0.090		
**PT**, >13 vs. ≤13 seconds	1.39(1.07-1.80)	**0.014**		
**PLT**, >100 vs. ≤100 ×10^9^/L	1.08(0.84-1.38)	0.543		
**NLR**, >2.4 vs. ≤2.4	1.82(1.49-2.23)	**<0.001**	1.51(1.21-1.88)	**<0.001**
**HBeAg**, positive vs. negative	1.26(1.02-1.56)	**0.034**		
**HBV-DNA**, >2000 vs. ≤2000 IU/mL	1.77(1.45-2.17)	**<0.001**	1.75(1.40-2.18)	**<0.001**
**Antiviral treatment**, Yes vs. No	0.85(0.67-1.07)	0.170		
**AFP**, >400 vs. ≤400 ng/mL	1.79(1.46-2.19)	**<0.001**	1.34(1.08-1.66)	**0.007**
**CEA**, >10 vs. ≤10 ng/mL	1.56(0.58-4.18)	0.376		
**Ca19-9**, >37 vs. ≤37 U/mL	1.33(1.06-1.68)	**0.015**		
**Intraoperative blood transfusion**, yes vs. no	1.41(1.08-1.83)	**0.010**		
**Surgical margin**, wide vs. narrow	0.47(0.38-0.59)	**<0.001**	0.62(0.49-0.78)	**<0.001**
**Tumor diameter**, cm	1.13(1.10-1.16)	**<0.001**	1.10(1.07-1.13)	**<0.001**
**Tumor number**, multiple vs. single	1.77(1.35-2.33)	**<0.001**	2.08(1.57-2.77)	**<0.001**
**Tumor capsule,** complete vs. incomplete	0.74(0.57-0.97)	**0.027**		
**Cirrhosis**, yes vs. no	1.00(0.82-1.22)	0.989		
**Edmondson-Steiner grade**, III-VI vs. I-II	2.03(1.19-3.46)	**0.010**		
**Postoperative TACE**, yes vs. no	0.71(0.58-0.87)	**0.001**	0.67(0.54-0.83)	**<0.001**

Bold values indicate statistical significance (P < 0.05). TBIL, total bilirubin; ALB, albumin; ALBI, albumin-bilirubin; ALT, Alanine aminotransferase; PT, Prothrombin time; PLT, platelet; NLR, neutrophil‐to‐lymphocyte ratio; HBV-DNA, hepatitis B virus-deoxyribonucleic acid; AFP, alpha fetoprotein; CEA, carcinoembryonic antigen; TACE, Transcatheter arterial chemoembolization.

**Table 3:** Univariate and multivariate cox regression analysis of OS in the training cohort.

Variable	Univariate analysis		Multivariate analysis	
	HR (95%CI)	*P*	HR (95%CI)	*P*
**Age**, >60 vs. ≤60 years	1.11(0.81-1.51)	0.534		
**Gender**, male vs. female	1.43(0.97-2.13)	0.074		
**Child-Pugh**, B vs. A grade	1.86(0.96-3.61)	0.067		
**TBIL**, >2000 vs. ≤2000 µmol/L	1.17(0.90-1.51)	0.231		
**ALB**, >35 vs. ≤35 g/L	0.62(0.41-0.92)	**0.018**		
**ALBI**,II vs. I grade	1.64(1.29-2.09)	**<0.001**		
**ALT**, >44 vs. ≤44 U/L	1.28(1.01-1.62)	**0.040**		
**PT**, >13 vs. ≤13 seconds	1.45(1.08-1.94)	**0.013**		
**PLT**, >100 vs. ≤100 ×10^9^/L	1.26(0.94-1.69)	0.129		
**NLR**, >2.4 vs. ≤2.4	2.20(1.75-2.77)	**<0.001**	1.83(1.41-2.37)	**<0.001**
**HBeAg**, positive vs. negative	1.29(1.01-1.64)	**0.041**		
**HBV-DNA**, >2000 vs. ≤2000 IU/mL	1.94(1.54-2.44)	**<0.001**	1.83(1.42-2.36)	**<0.001**
**Antiviral treatment**, Yes vs. No	0.69(0.52-0.92)	**0.012**		
**AFP**, >400 vs. ≤400 ng/mL	2.00(1.59-2.52)	**<0.001**	1.48(1.16-1.90)	**0.002**
**CEA**, >10 vs. ≤10 ng/mL	1.89(0.70-5.08)	0.206		
**Ca19-9**, >37 vs. ≤37 U/mL	1.36(1.04-1.78)	**0.026**		
**Intraoperative blood transfusion**, yes vs. no	1.83(1.38-2.42)	**<0.001**		
**Surgical margin**, wide vs. narrow	0.38(0.29-0.49)	**<0.001**	0.50(0.37-0.66)	**<0.001**
**Tumor diameter**, cm	1.15(1.12-1.18)	**<0.001**	1.09(1.06-1.13)	**<0.001**
**Tumor number**, multiple vs. single	1.72(1.26-2.34)	**0.001**	2.18(1.57-3.03)	**<0.001**
**Tumor capsule,** complete vs. incomplete	0.73(0.53-0.99)	**0.046**		
**Cirrhosis**, yes vs. no	1.00(0.79-1.26)	0.992		
**Edmondson-Steiner grade**, III-VI vs. I-II	2.26(1.16-4.40)	**0.016**		
**Postoperative TACE**, yes vs. no	0.60(0.47-0.76)	**<0.001**	0.58(0.45-0.74)	**<0.001**

Bold values indicate statistical significance (P < 0.05). OS, overall survival; TBIL, total bilirubin; ALB, albumin; ALBI, albumin-bilirubin; ALT, Alanine aminotransferase; PT, Prothrombin time; PLT, platelet; NLR, neutrophil‐to‐lymphocyte ratio; HBV-DNA, hepatitis B virus-deoxyribonucleic acid; AFP, alpha fetoprotein; CEA, carcinoembryonic antigen; TACE, Transcatheter arterial chemoembolization.

### Predictive ability of the nomograms in both cohorts

**Figure 2:**
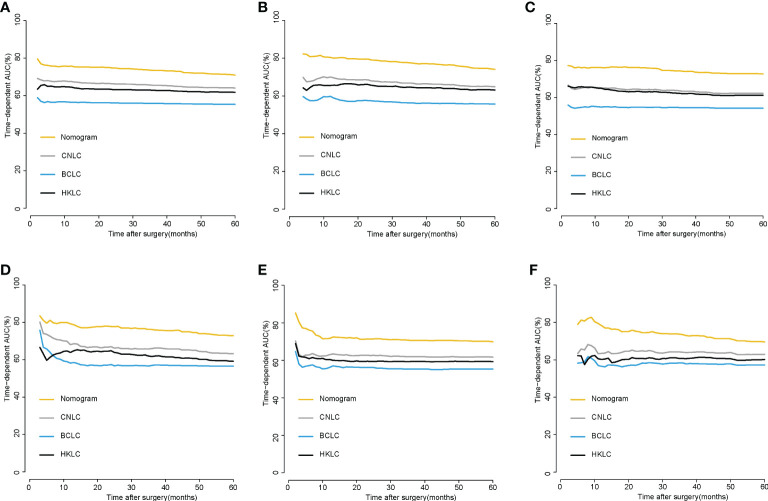
Time-dependent AUC of using the nomogram to predict recurrence and OS probability within 5 years in the training cohort **(A, B)**,internalvalidation cohorts **(C, D)** and external validation cohorts **(E, F)**.

**Figure 3:**
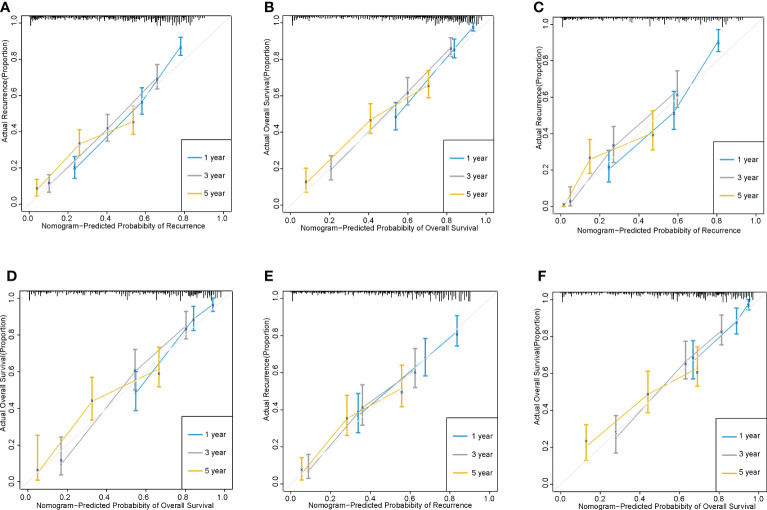
The calibration curves of recurrence and OS based on nomogram prediction and actual observation. 1-, 3- and 5-year recurrence and overall survival in the training cohorts **(A, B)**; 1-, 3- and 5-year recurrence and overall survival in the internal validation cohorts **(C, D)**;1-, 3- and 5-year recurrence and overall survival in the external validation cohorts **(E, F)**.

For the prediction of recurrence, the Harrell’s C-indices in the training and validation cohorts were 0.722 (95% CI, 0.711-0.732) and 0.747 (0.733-0.760), respectively, while the C-indices for OS were 0.759 (95% CI, 0.747-0.771) and 0.758 (0.741-0.776), respectively. In the external validation cohort, Harrell’s C-indices for recurrence and OS prediction were 0.719 (95% CI, 0.701-0.738) and 0.714(95% CI, 0.694-0.734), respectively. The C-index for predicting recurrence for the staging system CNLC, BCLC and HKLC were 0.639 (95% CI, 0.627-0.651),0.552 (95% CI, 0.542-0.562) and 0.617(95% CI, 0.604-0.629), respectively; the corresponding C-index values for predicted OS were 0.653 (95% CI,0.639-0.666),0.558 (95% CI,0.546-0.569) and 0.636(95% CI, 0.622-0.650) respectively. The time-dependent AUCs were >0.7 for predicting recurrence and OS within five years in three groups (**Figure 2**). Furthermore, the calibration curves for recurrence and OS at 1-, 3-, and 5- years indicated consistency between the nomogram predictions and actual data (**Figure 3**).

### Risk stratification using the nomograms

**Figure 4:**
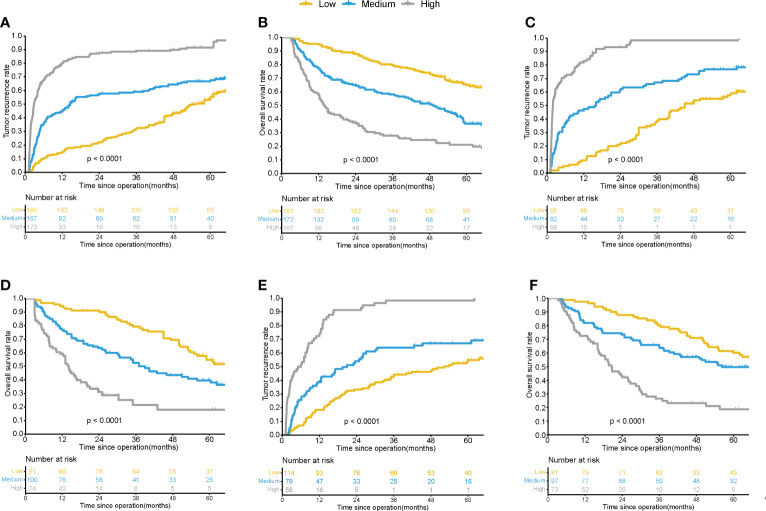
Kaplan-Meier survival curves for patients with different risk of postsurgical recurrence and OS by the nomogram score in training cohorts **(A, B)**, internalvalidation cohorts **(C, D)** and external validation cohorts **(E, F)**.

X-tile software was applied for the determination of the optimum cut off values of the cumulative scores of the nomograms. In the recurrence nomogram in the training cohort, patients were graded as low (≤ 172 points), intermediate (172-203 points), and high (>203 points) risk, with three-year recurrence rates of 32.1%, 59.1%, and 89.1%, respectively (P<0.001) (**Figure 4A**). In the OS nomogram, patients were graded as low (≤ 214 points), intermediate (214-246 points), and high (> 246 points) risk, with three-year OS rates of 84.8%, 59.1%, and 21.7%, respectively (P<0.001) (**Figure 4B**). The findings for the internal validation cohort and external validation cohort were consistent (**Figures 4C–F**).

### Webserver development for predicting prognosis

To make the nomograms more accessible, we have established a webserver allowing researchers and clinicians to predict recurrence and OS in HBV-associated MVI-positive HCC patients. This is available at https://baishileiehbh.shinyapps.io/HBV-MVI-HCC-RFS/ and https://baishileiehbh.shinyapps.io/HBV-MVI-HCC-OS/.

## Discussion

In East Asia, 70%-90% of HCC is caused by HBV infection, and the presence of MVI can be detected in 15.0%-57.1% of pathological specimens of HCC patients undergoing LR or LT (2, 6). There are various factors affecting the long-term prognosis of HBV-associated MVI-positive HCC patients, but previous staging guidelines often fail to take into account all relevant parameters to make individualized prognosis predictions for this type of HCC patients (6, 28, 29). In this paper, nomograms incorporate seven variables related to recurrence and OS in HBV-related MVI-positive HCC patients, encompassing tumor characteristics (tumor diameter, tumor number), inflammatory indicators (NLR), viral status (HBV-DNA), AFP, surgical status (surgical margins) and postoperative adjuvant therapy (postoperative adjuvant TACE). We established the first nomograms based on large sample data to predict recurrence and OS after LR in HBV-associated MVI-positive HCC patients. The C-indices of nomograms for predicting recurrence and OS were 0.722 and 0.759, respectively, for the training cohort with good predictive ability and better predictive ability than BCLC staging and CNLC staging.

Several preoperative high-risk indicators such as HBV-DNA and NLR have been found to be linked with reduced survival after LR (29–31). Li et al. observed that preoperative HBV-DNA > 2000 IU/ml was linked with a higher MVI incidence and a greater likelihood of early recurrence in HCC patients (28). Elevated NLR has also been shown to reduce the OS after LR in HCC patients (30). AFP, tumor diameter, and tumor number are well-known indicators affecting outcomes after LR for HCC and were included in several clinical guidelines (2, 22). Here, we also fully considered the effects of these factors.

Surgical margins are known to influence the prognosis of HCC after LR (23, 29, 32, 33). Portal vein infiltration and small metastatic nodules are usually seen within a 10-mm distance of the primary tumor and rarely at distances over 20 mm (34–36). Thus, a minimum resection margin of 10 mm is recommended. However, for tumors adjoining large blood vessels or in the presence of poor liver function, narrow resection may be a more appropriate surgical approach because it preserves more liver tissue and avoids damage to major blood vessels, but it also leads to a greater likelihood of recurrence and reduced survival. Yang et al. analyzed 929 patients with HBV-related MVI-positive HCC patients, including 545 patients with narrow margins and 545 patients with wide margins, the 5-year recurrence and OS rates were 71.1% vs. 85.9% and 44.9% vs. 25.0% for wide and narrow margin patients, respectively (both P<0.001) (29). In this article, it was also demonstrated that for HBV-related MVI-positive HCC patients, a wide margin surgical approach improves patient prognosis compared to a narrow margin.

There is no consensus on the use or type of adjuvant therapy for MVI-positive HCC. TACE involves the injection of embolic and chemotherapeutic drugs into arteries to decrease the tumor blood supply and induce ischemic necrosis. Recent studies have also shown that postoperative adjuvant TACE prolongs both recurrence-free survival (RFS) and OS in patients with HCC, especially in MVI-positive patients (37–39). The randomized clinical trial by Wei et al. included 250 patients with MVI-positive HCC, including 125 each with and without postoperative adjuvant TACE, respectively, observing that the postoperative TACE markedly increased both RFS and OS in MVI-positive patients (39). In this paper, 342 of 795 patients were treated with postoperative adjuvant TACE, and the multivariate analysis also indicated that postoperative adjuvant TACE treatment was a protective factor for recurrence and OS, confirming the previous study.

There are several limitations to this study. First, the study was conducted at a single institution and needs confirmation by multicenter results. Second, the nomograms are specific for HBV-infected HCC patients and are not applicable to patients with HCC due to other etiologies. Third, we did not exclude patients with HBV who also had other etiologies such as alcoholism or fatty liver, etc., this may have a certain impact on the research results. Lastly, the study is retrospective, leading to inevitable issues in selection bias and bias from incomplete adherence to the post-operative follow-up protocol.

In summary, we developed and validated nomograms for predicting one-, three-, and five-year recurrence and OS in HBV-associated MVI-positive HCC. The nomograms were found to have good predictive ability and could accurately differentiate between patients differing in recurrence and survival risk. It is recommended that close monitoring together with adjuvant therapy be used for patients at risk of tumor recurrence following surgery.

## Data availability statement

The data that support the findings of this study are available from the corresponding author upon reasonable request. Requests to access these datasets should be directed to Shilei Bai, 504739426@qq.com.

## Ethics statement

The studies involving human participants were reviewed and approved by Eastern Hepatobiliary Surgery Hospital ethics committee. The patients/participants provided their written informed consent to participate in this study.

## Author contributions

Study concept and design: SB, PY, YW, YT and KW. Acquisition, analysis, or interpretation of data: SB, AS, PY and FS. Statistical analysis: SB, JW, CL, YX. Critical revision of the manuscript for important intellectual content: YT and KW. All authors contributed to the article and approved the submitted version.

## Supplementary material

Supplementary Figure 1 Flow Chart of patient’s inclusion.

Supplementary Figure 2 Postoperative recurrence **(A)** and OS **(B)** in the training and validation cohorts.
