# Supplementary figures and images for: Development and validation of prognostic dynamic nomograms for hepatitis B Virus-related hepatocellular carcinoma with microvascular invasion after curative resection
Source: Front Oncol. 2023 Apr 19;13:1166327. doi: 10.3389/fonc.2023.1166327 (PMC10154689; doi:10.3389/fonc.2023.1166327)

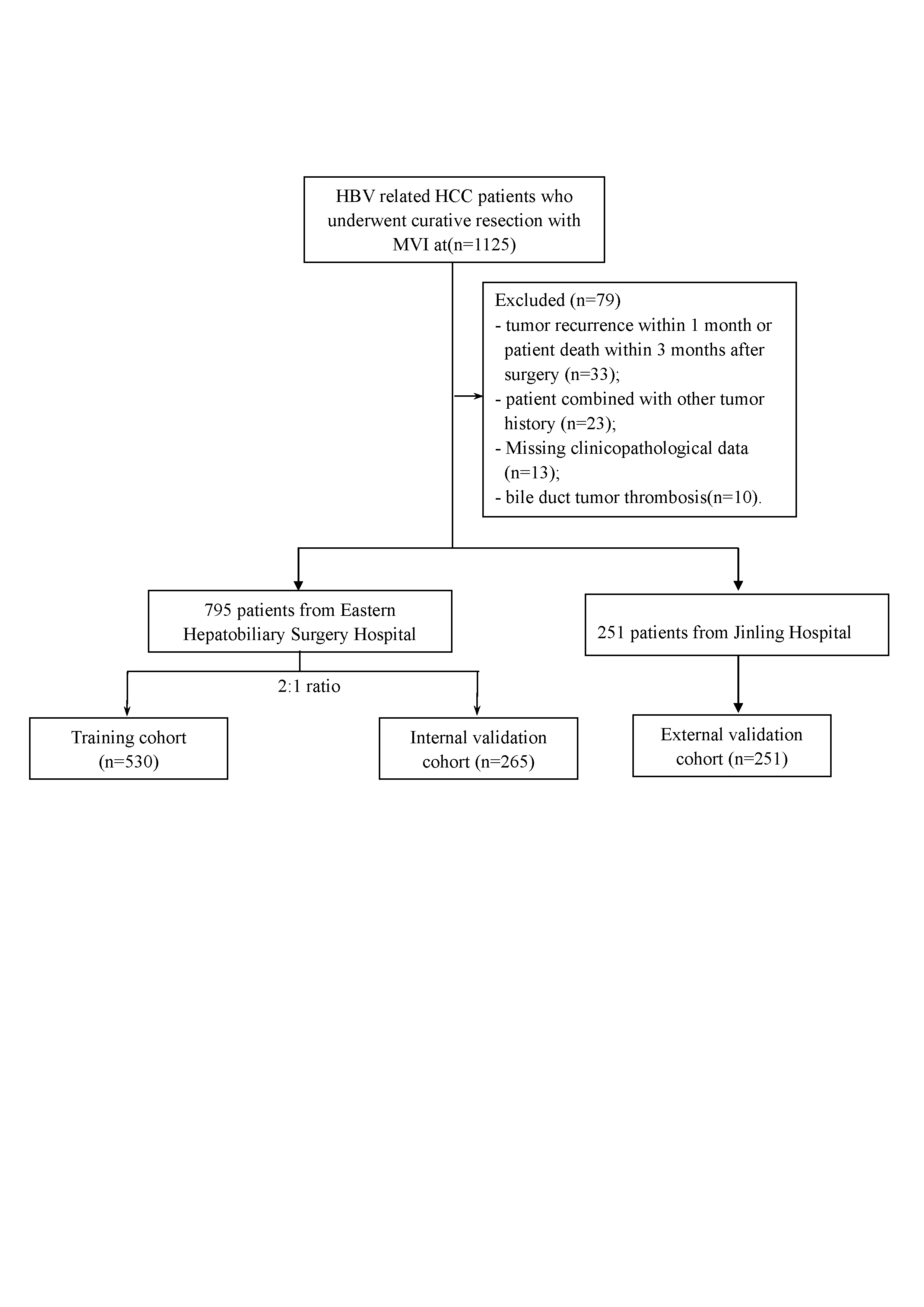

Supplement: Supplementary Figure 1 — Flow Chart of patient’s inclusion. [file Image_1.tif]

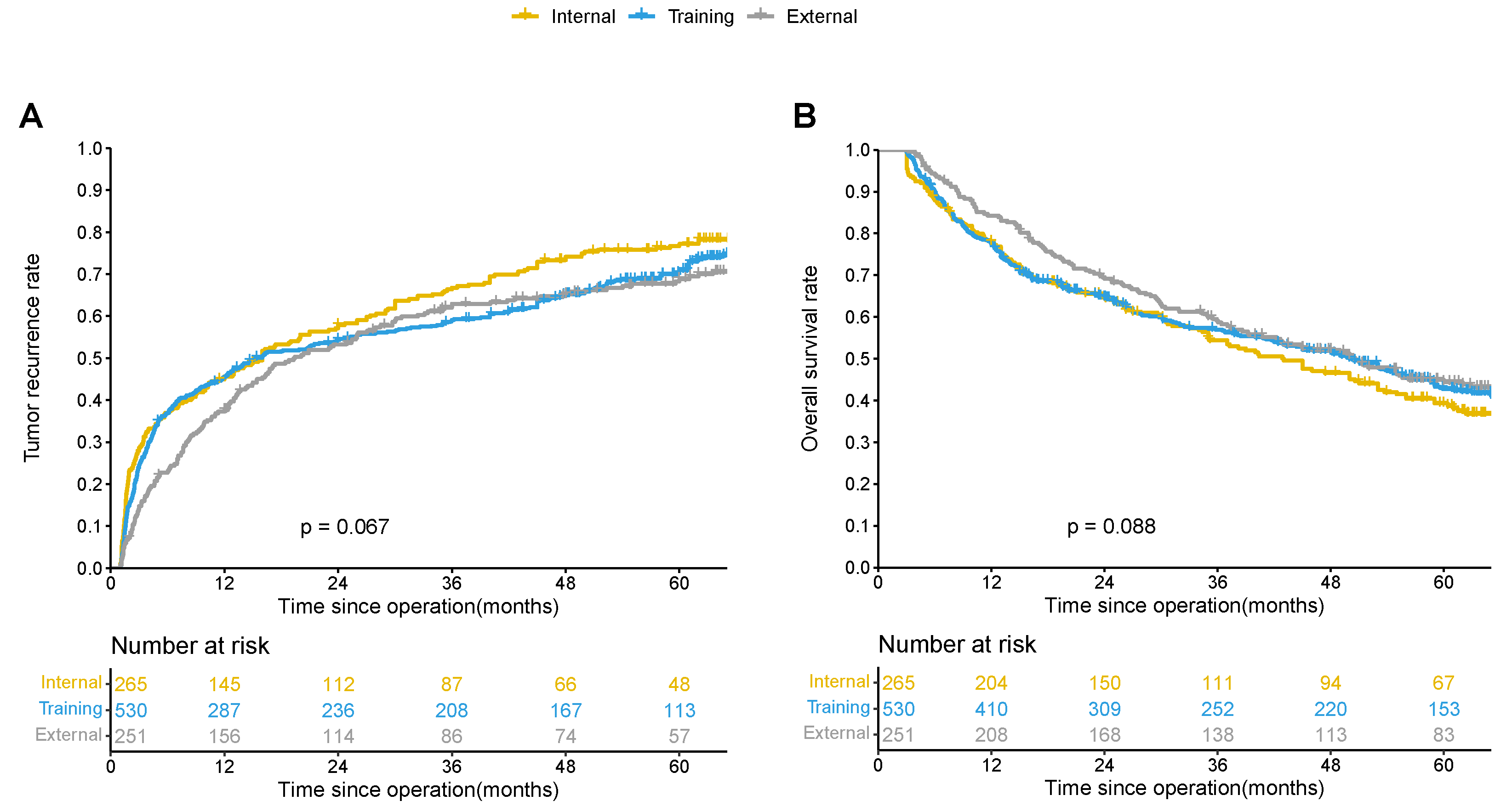

Supplement: Supplementary Figure 2 — Postoperative recurrence (A) and OS (B) in the training and validation cohorts. [file Image_2.tif]
